# Comparative chloroplast-specific SNP and nSCoT markers analysis and population structure study in kiwifruit plants

**DOI:** 10.1186/s41065-024-00321-3

**Published:** 2024-05-17

**Authors:** Yinling Ding, Yu Wang, Zhe Chen, Jiamin Dou, Yihao Zhang, Yu Zhang

**Affiliations:** 1 School of Biological Science and Engineering, Shaanxi University of Technology, Hanzhong, Shaanxi Province China; 2 Shaanxi Province Key Laboratory of Bio-resources, Hanzhong, Shaanxi Province China; 3 QinLing-Bashan Mountains Bioresources Comprehensive Development C. I. C, Hanzhong, Shaanxi Province China; 4 Qinba State Key Laboratory of biological resources and ecological environment,, Hanzhong,, Shaanxi Province China; 5 Shaanxi Fruit Industry Group Limited Hanzhong Kiwifruit R&D Centre, Hanzhong, Shaanxi Province China

**Keywords:** *Actinidia*, Chloroplast-specific SNP, Chloroplast-specific DNA haplotype diversity, nSCoT, Population structure

## Abstract

**Background:**

Kiwifruit (Actinidiaceae family) is an economically important fruit tree in China and New Zealand. It is a typical dioecious plant that has undergone frequent natural hybridization, along with chromosomal ploidy diversity within the genus *Actinidia*, resulting in higher genetic differences and horticultural diversity between interspecific and intraspecific traits. This diversity provides a rich genetic base for breeding. China is not only the original center of speciation for the *Actinidia* genus but also its distribution center, housing the most domesticated species: *A. chinensis* var. *chinensis*, *A. chinensis* var. *deliciosa*, *A. arguta*, and *A. polygama*. However, there have been relatively few studies on the application of DNA markers and the genetic basis of kiwifruit plants. By combining information from chloroplast-specific SNPs and nuclear SCoT (nSCoT) markers, we can uncover complementary aspects of genetic variation, population structure, and evolutionary relationships. In this study, one chloroplast DNA (cpDNA) marker pair was selected out of nine cpDNA candidate pairs. Twenty nSCoT markers were selected and used to assess the population structure and chloroplast-specific DNA haplotype diversity in 55 kiwifruit plants (*Actinidia*), including 20 samples of *A. chinensis* var. *chinensis*, 22 samples of *A. chinensis* var. *deliciosa*, 11 samples of *A. arguta*, and two samples of *A. polygama*, based on morphological observations collected from China.

**Results:**

The average genetic distance among the 55 samples was 0.26 with chloroplast-specific SNP markers and 0.57 with nSCoT markers. The Mantel test revealed a very small correlation (*r* = 0.21). The 55 samples were categorized into different sub-populations using Bayesian analysis, the Unweighted Pair Group Method with the Arithmetic Mean (UPGMA), and the Principal Component Analysis (PCA) method, respectively. Based on the analysis of 205 variable sites, a total of 15 chloroplast-specific DNA haplotypes were observed, contributing to a higher level of polymorphism with an Hd of 0.78. Most of the chloroplast-specific DNA haplotype diversity was distributed among populations, but significant diversity was also observed within populations. H1 was shared by 24 samples, including 12 of *A. chinensis* var. *chinensis* and 12 of *A. chinensis* var. *deliciosa*, indicating that H1 is an ancient and dominant haplotype among the 55 chloroplast-specific sequences. H2 may not have evolved further.The remaining haplotypes were rare and unique, with some appearing to be exclusive to a particular variety and often detected in single individuals. For example, the H15 haplotype was found exclusively in *A. polygama*.

**Conclusion:**

The population genetic variation explained by chloroplast-specific SNP markers has greater power than that explained by nSCoTs, with chloroplast-specific DNA haplotypes being the most efficient. Gene flow appears to be more evident between *A. chinensis* var. *chinensis* and *A. chinensis* var. *deliciosa*, as they share chloroplast-specific DNA haplotypes, In contrast, *A.arguta* and *A. polygama* possess their own characteristic haplotypes, derived from the haplotype of *A. chinensis* var. *chinensis*. Compared with *A. chinensis*, the *A.arguta* and *A. polygama* showed better grouping. It also seems crucial to screen out, for each type of molecular marker, especially haplotypes, the core markers of the *Actinidia* genus.

## Introduction

Kiwifruit is highly regarded for its nutritional and medicinal value, and it has become one of the successfully domesticated fruit trees of the 20th century, the cultivation area of kiwifruit plants is increasing year by year in China, but the basic genetic research, especially at the DNA level, is still weak compared to other crops [1], and in-depth exploration of these issues is essential for the sustainable development of the kiwifruit industry.

DNA molecular markers, characterized by their high polymorphism and stable performance, are increasingly employed in various aspects, such as taxonomy [2], population structure analysis [3], genetic diversity assessment [4], breeding [5], chromosome ploidy analysis [6], and genome-wide association studies [7]. Chloroplast DNA (cpDNA), known for its conserved and slow evolutionary rate, plays a crucial role in plant evolution [8], phylogeographic studies [9], classification [10], diversity assessment [11], population structure analysis [12], and breeding [13]. Among these, chloroplast SNPs (single nucleotide polymorphisms) and chloroplast-specific DNA haplotypes are newer types of molecular markers with the characteristics of SNP markers within the chloroplast genome [14–19].

Nuclear SCoT (nuclear start codon targeted polymorphism, nSCoT) markers, as highly effective nuclear DNA markers, offer numerous advantages, including the number of polymorphic bands (NPB), percentage of polymorphic bands (PPB), resolving power (Rp), effective multiplex ratio (EMR), and marker index (MI) [1]. To date, limited comparative studies have been reported on cpDNA and nuclear DNA markers, especially regarding haplotype analysis of chloroplast genomes, by studying haplotype diversity of kiwifruit plants, we can uncover important information about population history, migration patterns, and evolutionary dynamics.

To date, there has been a scarcity of comparative studies focusing on both chloroplast DNA (cpDNA) and nuclear DNA markers, as well as haplotype analysis of chloroplast genomes. Such research enables us to glean valuable insights into a wide array of aspects, including the population history, migration patterns, and evolutionary dynamics of kiwifruit plants. In this study, we constructed three types of molecular marker datasets based on chloroplast SNPs, chloroplast-specific DNA haplotypes, and nSCoT data. These datasets allowed us to perform comprehensive phylogenetic and population analyses.

## Materials and methods

### Plant materials

A total of 55 kiwifruit plant genotypes comprised 20 samples of A. chinensis var. chinensis, 22 samples of A. chinensis var. deliciosa, 11 samples of A.arguta and 2 samples of A. polygama, which were established based on morphological observations, representing most kiwifruit germplasm of China; the samples were collected from the kiwifruit experimental farm of different regions during the 2020 growing season (Table 1).

**Table 1:** List of 55 samples used in this study

Code	Varieties(lines)	Flesh color	Taxa	Code	Varieties(lines)	Flesh color	Taxa
1	Wild materials	Yellow	*A.chinensis var.chinensis*	29	Cuixiang	Green	*A.chinensis* var.*deliciosa*
2	Wild materials	Yellow	*A.chinensis var.chinensis*	30	Wild materials	Green	*A.chinensis* var.*deliciosa*
3	Jintao1	Yellow	*A.chinensis var.chinensis*	31	Wild materials	Green	*A.chinensis* var.*deliciosa*
4	Huayou	Yellow	*A.chinensis var.chinensis*	32	Xuxiang	Green	*A.chinensis* var.*deliciosa*
5	Huangjinguo	Yellow	*A.chinensis var.chinensis*	33	Wild materials	Green	*A.chinensis* var.*deliciosa*
6	Wild materials	Yellow	*A.chinensis var.chinensis*	34	Qinmei	Green	*A.chinensis* var.*deliciosa*
7	Nongdajinmi	Yellow	*A.chinensis var.chinensis*	35	Haiwode	Green	*A.chinensis* var.*deliciosa*
8	Qinhong1	Green	*A.chinensis var.chinensis*	36	Wild materials	Green	*A.chinensis* var.*deliciosa*
9	Qinhong2	Green	*A.chinensis var.chinensis*	37	Jinkui	Green	*A.chinensis* var.*deliciosa*
10	Nongdajinmi	Yellow	*A.chinensis var.chinensis*	38	Wild materials	Green	*A.chinensis* var.*deliciosa*
11	Wild materials	Yellow	*A.chinensis var.chinensis*	39	Wild materials	Green	*A.chinensis* var.*deliciosa*
12	Hongyang	Yellow	*A.chinensis var.chinensis*	40	Wild materials	Green	*A.chinensis* var.*deliciosa*
13	Qihong3	Green	*A.chinensis var.chinensis*	41	Xuxiang	Green	*A.chinensis* var.*deliciosa*
14	Huayou	Yellow	*A.chinensis var.chinensis*	42	Wild materials	Green	*A.chinensis* var.*deliciosa*
15	Wild materials	Yellow	*A.chinensis var.chinensis*	43	F1(*A.arguta*×*A.arguta*)	Green	*A.arguta*
16	Wild materials	Yellow	*A.chinensis var.chinensis*	44	F1(*A.arguta*×*A.arguta*)	Green	*A.arguta*
17	Jintao2	Yellow	*A.chinensis var.chinensis*	45	F1(*A.arguta*×*A.chinensis*)	Green	*A.arguta*
18	Hongyang	Yellow	*A.chinensis var.chinensis*	46	F1(*A.arguta*×*A.arguta*)	Green	*A.arguta*
19	Jinyan	Yellow	*A.chinensis var.chinensis*	47	F1(*A.arguta*×*A.arguta*)	Green	*A.arguta*
20	Wild materials	Green	*A.chinensis var.chinensis*	48	F1(*A.arguta*×*A.arguta*)	Green	*A.arguta*
21	Jinkui	Green	*A.chinensis* var.*deliciosa*	49	F1(*A.arguta*×*A.arguta*)	Green	*A.arguta*
22	Wild materials	Green	*A.chinensis* var.*deliciosa*	50	*A.arguta*♀	Green	*A.arguta*
23	Nongdamixiang	Green	*A.chinensis* var.*deliciosa*	51	*A.arguta*♀	Green	*A.arguta*
24	Nongdayuxiang	Green	*A.chinensis* var.*deliciosa*	52	*A.arguta*♂2		*A.arguta*
25	Wild materials	Green	*A.chinensis* var. *delicios*	53	Wild materials	Green	*A.arguta*
26	Wild materials	Green	*A.chinensis* var.*deliciosa*	54	Shuiyangtao	Yellow	*A. Polygama*
27	Cuixiang	Green	*A.chinensis* var.*deliciosa*	55	Shuiyangtao	Yellow	*A. Polygama*
28	Qinmei	Green	*A.chinensis* var.*deliciosa*				

### DNA extraction and marker genotyping

Genomic DNA was extracted from fresh leaves of each individual using the modified CTAB technique and detected with 0.8% agarose gel electrophoresis. PCR was carried out as follows: 2×Taq Master Mix (7.5 µL), forward and reverse primers 1 µL each (2 µL for ScoT primers), Rnase-free water (3.5 µL), and kiwifruit genomic DNA (2 µL). The reactions were programed as follows: initial denaturation at 94.0 °C for 5 min, denaturation at 94.0 °C for 1 min, annealing at 44 –62 °C for 1 min, and extension at 72.0 °C for 1 min, for a total of 35 cycles. The duration of extension was 10 min; then storage at 4.0 °C. The selected primers were synthesized by the Shanghai Sengon Biological Engineering Technology and Service Company (Shanghai, China). Initially, six germplasms (three samples of A. chinensis var. chinensis, two samples of A. chinensis var. deliciosa and one sample of A.arguta) were used to screen cpDNA markers for high polymorphim. Then, one pair of clear and highly polymorphic cpDNA and 20 pairs of nSCoT marker primers were selected from 9 cpDNA markers and 50 nSCoT pairs, respectively. Electrophoresis was performed using 8% non-denaturing polyacrylamide gel under 160 V voltage; the bands were visualized via silver staining. Illumina paired-end sequencing of PCR products-cpDNA was undertaken by Beijing Aoke Biotechnology Co., Ltd. (Beijing, China).

### nSCoT marker efficiency analysis

For nScoT markers following electrophoresis, each amplification band corresponded to a primer hybridization locus and was considered as an effective molecular marker. Each polymorphic band detected by a same given primer represented an allelic mutation. In order to generate molecular data matrices, clear bands for each fragment were scored in every accession for each primer pair and recorded as 1 (presence of a fragment), 0 (absence of a fragment), and 9 (complete absence of band ). The value of the polymorphism information content (PIC) was calculated using the PIC_Calc 0.6 program (http://www.Bio-soft.Net/dna/pic.Htm). The value of PIC varied from 0 to 1, with 0 indicating an absence of polymorphism at a given locus and 1 reflecting multiple alleles at a given locus. The level of polymorphism of each marker was assessed by the polymorphism information content [20], which measures the extent of genetic variation: PIC values smaller than 0.25 indicate low levels of polymorphism associated to a locus, PIC values between 0.25 and 0.5 imply moderate levels of polymorphism, while PIC values greater than 0.5 indicate high levels of polymorphism.

### Correlation analysis between genetic distance matrices

Correlation analysis between genetic distance matrices was conducted by the Mantel test in the GenAIEx6.3 software.

### Genetic constitution analysis

Bayes clustering analysis by the STRUCTURE v2.3.4 software was used to simulate population genetic structure. Using a membership probability threshold of 0.60, population K values from 1 to 5 were simulated with 20 iterations for each K using 10,000 burn-in periods followed by 10,000 Markov Chain Monte Carlo iterations in order to obtain an estimate of the most probable number of population. Delta K was plotted against K values; the best number of clusters was determined following the method proposed by Evanno et al. [21] and obtained via the Structure Harvester platform (http://taylor0.biology.ucla.edu/structureHarvester/). Genetic distances were computed using the JC method of the NTSYS-pc2.10e and MEGA X [22] software based on chloroplast-specific SNP and nSCoT markers, respectively, and the cluster analysis were conducted using the UPGMA method by MEGA X, and the principal component analysis was conducted under NTSYS-pc2.10e [23].

### Chloroplast-specific DNA haplotype diversity and network analysis

Chloroplast-specific DNA haplotype diversity and network analyses were conducted in MEGA X and DnaSPV6.12.03 (http:/www.ub.es/dnasp) [24], in which the evolutionary history was inferred using the Maximum Likelihood method and the Tamura-Nei model. Haplotype network construction was carried out by using Maximum Parsimony (MP) by Network. Ancestral states were inferred using the Maximum Likelihood method and the Tamura-Nei model [25, 26]. The tree shows a set of possible nucleotides (states) at each ancestral node based on their inferred likelihood at site 1. The set of states at each node is ordered from most likely to least likely, excluding states with probabilities below 5%.

## Results

### Marker efficiency analysis

**Fig. 1:**
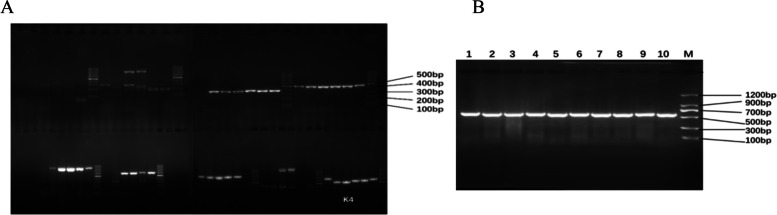
Screening for cpDNA markers. **A** The result of PCR for nine cpDNA markers. **B** The partial result of PCR by k4 primer

In this study, we initially utilized six germplasms, comprising three samples of A. chinensis var. chinensis, two samples of A. chinensis var. deliciosa, and one sample of A. arguta, to screen for highly polymorphic markers from a pool of nine chloroplast DNA (cpDNA) markers and 50 nuclear Start Codon Targeted (nSCoT) markers. Subsequently, we narrowed down our selection to K4 and 20 nSCoT markers for further investigation (as illustrated in Fig. 1; Tables 2 and 3). The fragment sizes amplified using the K4 marker ranged from 475 bp to 760 bp. A total of 146 bands were generated with the 20 nSCoT markers across the 55 kiwifruit samples, of which 139 were identified as polymorphic bands, resulting in a Percentage of Polymorphic Bands (PPB) of 95.21% (as shown in Table 3).

**Table 2:** Information of cpDNA markers

Title	Primer(5’-3’)	Primer(5’-3’)	Tm ℃
ITS	AgAAgTCgTAACAAggTTTCCgTAgg	TCCTCCgCTTATTgATATgC	58
rpL16	gCTATgCTTAgTgTgTgACTCgTTg	CCCTTCATTCTTCCTCTATgTTg	62.3
3 − 1(KIM)	CgTACAgTACTTTTgTgTTTACgAg	ACCCAgTCCATCTggAAATCTTggTTC	59.3
390–1326	CgATCTATTCATTCAATATTTC	TCTAgCACACgAAAgTCgAAgT	51.3
X-5	TAATTTACgATCAATTCATTC	gTTCTAgCACAAgAAAgTCg	48.3
1–8	ATgTATCAACAgAATCRT	AAAgTTCTAgCACAAgAAAgTCgA	44
K2	CggTggTTTggTTTCCTAgC	AgTTCAATAgCTgCATTgTCC	62
K4	ggCACTTgggATCCTATgg	CCCCAAATgCTACgggAATg	60
psbA-trnH	gTTATgCATgAACgTAATgCTC	CgCgCATggTggATTCACAAATC	56.3

**Table 3:** Amplification results of nSCoT primers

Primer name	Sequence(5’-3’)	Total number of bands (TNB)	The number of polymorphic bands(NPB)	Percentage of polymorphic bands(PPB)%	Polymorphism information content(PIC)
nSCoT1	CAACAATGGCTACCACCA	10	10	100	0.82
nSCoT2	CAACAATGGCTACCACCC	7	7	100	0.74
nSCoT3	CAACAATGGCTACCACCG	7	7	100	0.75
nSCoT4	CAACAATGGCTACCACCT	13	13	100	0.83
nSCoT5	CAACAATGGCTACCACGA	10	7	70	0.80
nSCoT6	CAACAATGGCTACCACGC	9	9	100	0.81
nSCoT7	CAACAATGGCTACCACGG	9	7	77.78	0.78
nSCoT8	CAACAATGGCTACCACGT	4	4	100	0.65
nSCoT9	CAACAATGGCTACCAGCA	7	5	71.43	0.63
nSCoT10	CAACAATGGCTACCAGCC	9	9	100	0.80
nSCoT11	AAGCAATGGCTACCACCA	5	5	100	0.67
nSCoT12	ACGACATGGCGACCAACG	9	9	100	0.82
nSCoT13	ACGACATGGCGACCATCG	9	9	100	0.81
nSCoT14	ACGACATGGCGACCACGC	7	7	100	0.71
nSCoT15	ACGACATGGCGACCGCGA	7	7	100	0.75
nSCoT16	ACCATGGCTACCACCGAC	6	6	100	0.59
nSCoT17	ACCATGGCTACCACCGAG	3	3	100	0.55
nSCoT18	ACCATGGCTACCACCGCC	6	6	100	0.72
nSCoT19	ACCATGGCTACCACCGGC	4	4	100	0.50
nSCoT20	ACCATGGCTACCACCGCG	5	5	100	0.68

### Correlation analysis between genetic distance matrices

**Fig. 2:**
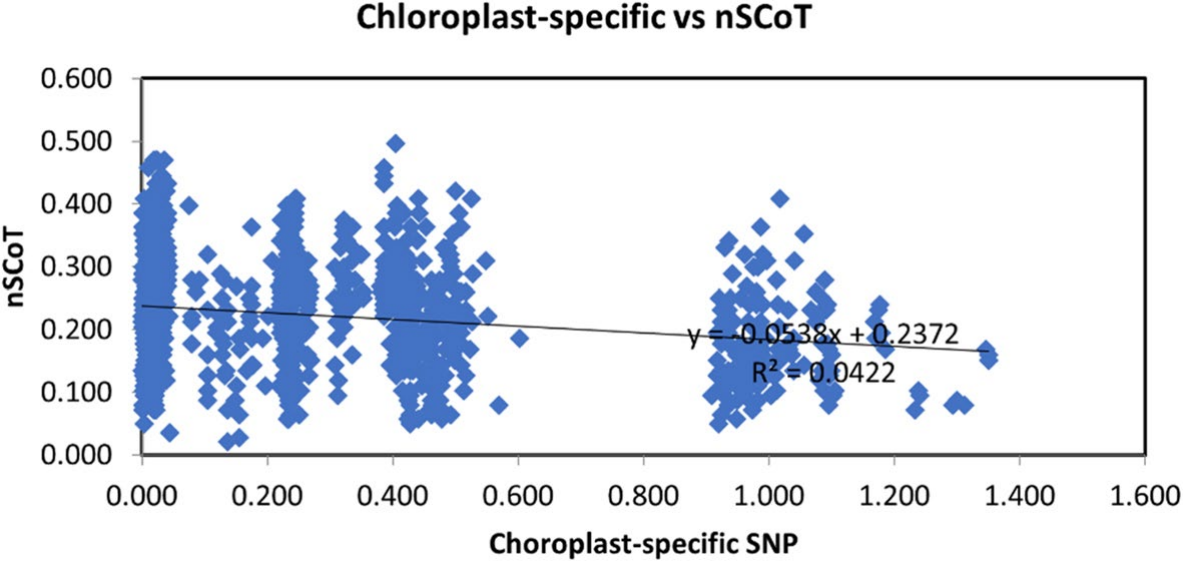
The correlation between the genetic distance matrices based on chloroplast-specific SNP and nSCoT markers

To assess the relationship between the genetic distance matrices generated by the nSCoT and chloroplast-specific SNP molecular markers, Mantel tests [25] were employed. The Mantel tests used the following correlation thresholds: *R* ≥ 0.9 indicated a significant correlation, 0.8 ≤ *r* < 0.9 denoted a moderate correlation, 0.7 ≤ *r* < 0.8 represented a weak correlation, and *r* < 0.7 signified no correlation. The analysis revealed a correlation coefficient of 0.21 (as depicted in Fig. 2), indicating no significant correlation between the genetic distance matrices. This result may be attributed to the utilization of different numbers and types of markers in the study.

### Genetic constitution analysis

#### Bayes clustering analysis

**Fig. 3:**
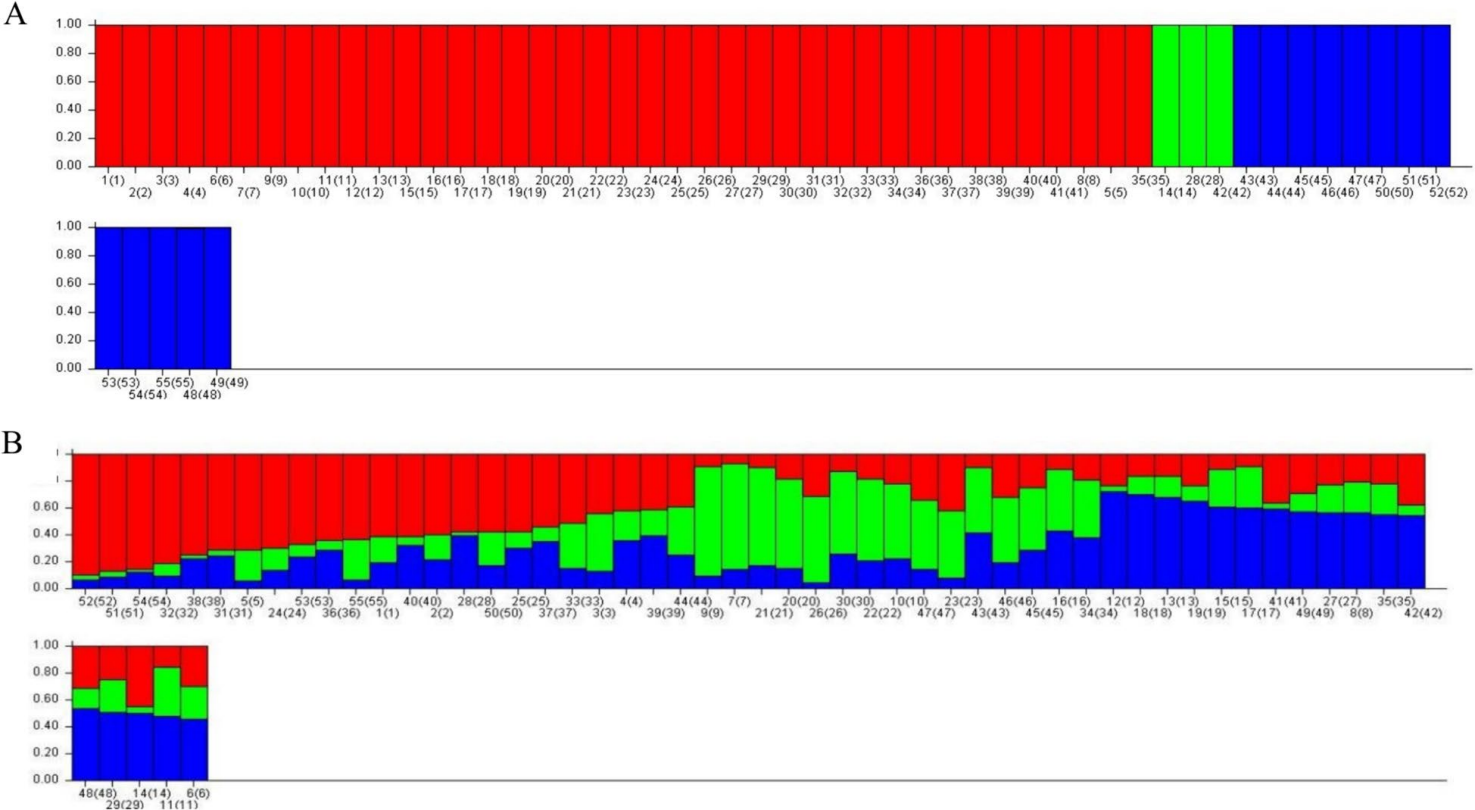
Bayes clustering analysis of the number of population for K. The number of subpopulations (k) was identified based on maximum likelihood and k values. The most likely value of k identified by STRUCTURE software were both observed at k=3 based on nSCoT markers (**A**) and chloroplast-specific SNP markers (**B**). Note: Red bands: Group 1, Green bands: Group 2, Blue bands: Group 3. The proportion of each color reflects the probability that each of the test materials (numbered from 1 to 55) belongs the corresponding group

To investigate the Genetic constitution within 55 kiwifruit genotypes, we utilized a dataset consisting of 146 polymorphic bands and 696 SNPs with fragment sizes ranging from 475 bp to 760 bp. We observed that the Delta K values reached their maximum at K = 3 for both the nSCoT and chloroplast-specific SNP markers, as indicated in Fig. 3. This finding suggests that the 55 kiwifruit germplasms are best classified into three distinct subgroups.

#### UPGMA clustering analysis

**Fig. 4:**
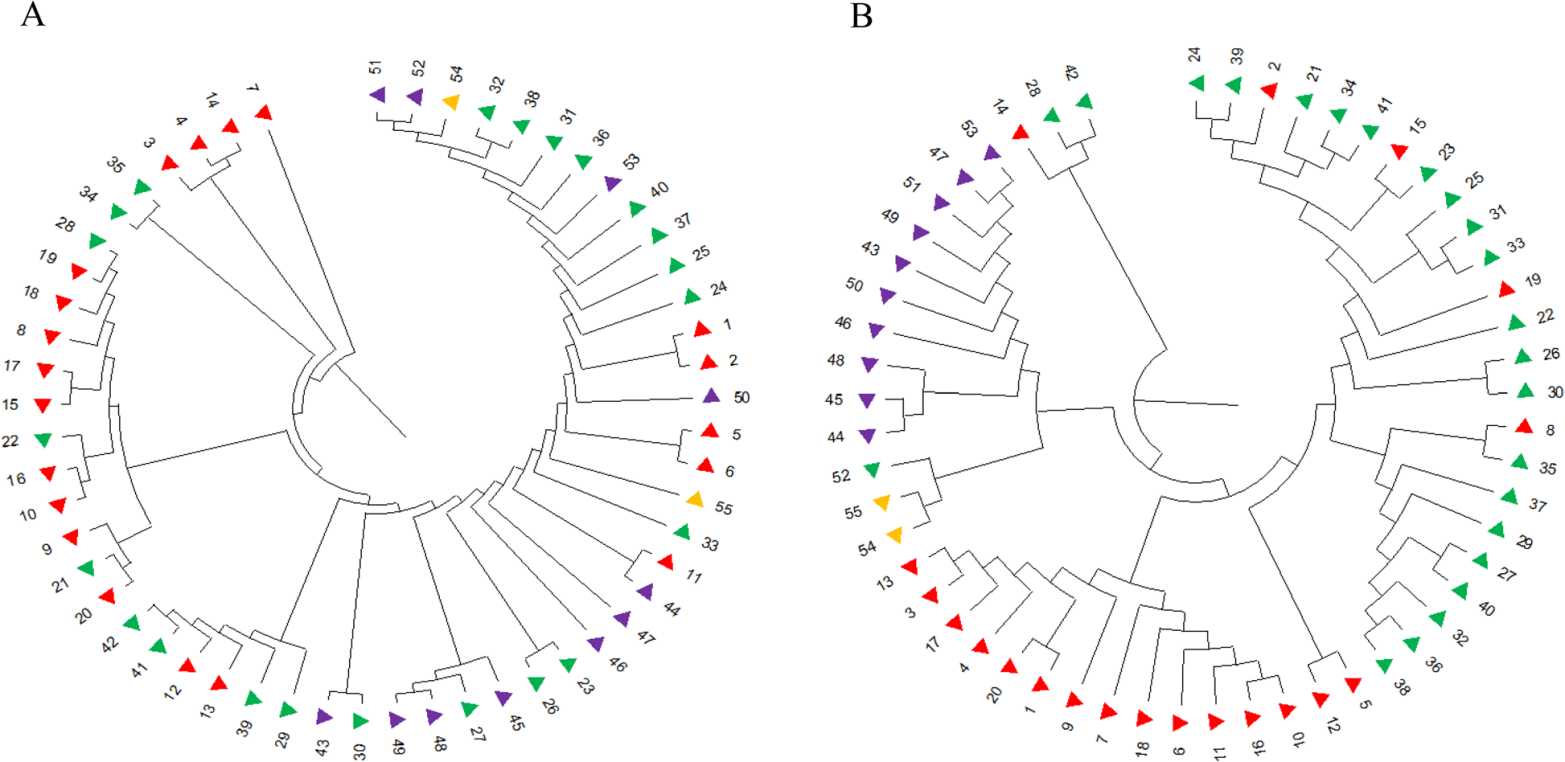
Cluster dendrogram of 55 kiwifruit genotypes constructed by UPGMA. **A** based on nSCoT markers; **B** based on chloroplast-specific SNP markers. Note: The red numbers represent *A. chinensis* var. *chinensis*, the green numbers represent *A. chinensis* var. *deliciosa*, the purple numbers represent *A.arguta* and the apricot numbers represent *A. polygama*

A dendrogram was constructed through cluster analysis using the unweighted pair-group method with arithmetic means (UPGMA), which demonstrated that the 55 genotypes were divided into two distinct groups, as illustrated in Fig. 4. For nSCoT markers, Group I consisted of a single variety, while Group II included the remaining 54 varieties. The average genetic distance within this dataset was calculated at 0.57. Notably, the two most closely related materials were identified as samples 51 and 52, with a genetic distance of just 0.02. This closeness indicates a strong genetic relationship between these two samples, with a sister line connecting the female and male plants. In contrast, when considering chloroplast-specific SNP markers, the 55 genotypes were divided into three and 52 varieties in Groups I and II, respectively. In this context, samples with closer kinship demonstrated a tendency to cluster together effectively. Examples of this included samples 3 and 13, 9 and 16, 15 and 23, 34 and 41, and 47 and 53, all of which exhibited a genetic distance of 0, highlighting their strong genetic relatedness.

#### Principal components analysis

The top three principal components were used to analyze population structure. The results showed that the three PCs based on nSCoT markers had contribution rates of 28.42%, 15.33% and 12.86%, which indicated inconspicuous grouping (Fig. 4A), In contrast, the three principal components derived from chloroplast-specific SNP markers demonstrated significantly higher contribution rates of 80.71%, 22.98%, and 1.89%. These components clearly segregated the 55 genotypes into two major groups, as shown in Fig. 4B.

**Fig. 5:**
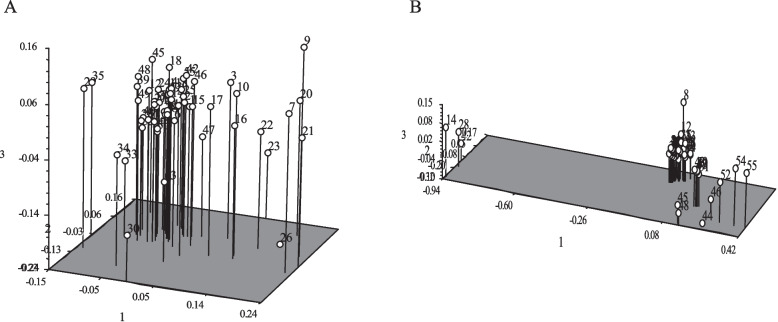
PCA plots based on the first three components. **A** based on nSCoT markers; **B** based on chloroplast-specific SNP markers

The analysis performed using Bayes, UPGMA and PCA clustering yielded dissimilar results, in which clustering the 55 genotypes into 3 sub-populations was based on Bayes clustering. Of note, clustering results based on chloroplast-specific SNP using three different methods had good consistency with previous results from STRUCTURE (Fig. 5).

### Chloroplast-specific DNA haplotype diversity and network

In this study encompassing 55 genotypes, the fragment sizes amplified by K4 primers ranged from 475 bp to 760 bp. Since the results of haplotype diversity analysis are influenced by the number of variable sites relative to the total number of sites, we selected specific fragment lengths of 734 bp, 674 bp, 574 bp, and 474 bp, each possessing 205, 205, 185, and 130 variable sites, respectively, for haplotype diversity analysis. This approach allowed us to identify the presence of 15, 15, 14, and 12 haplotypes, corresponding to the different fragment lengths, and the values of haplotype diversity (Hd) for these fragment lengths were 0.78, 0.78, 0.77, and 0.77, respectively.

**Fig. 6:**
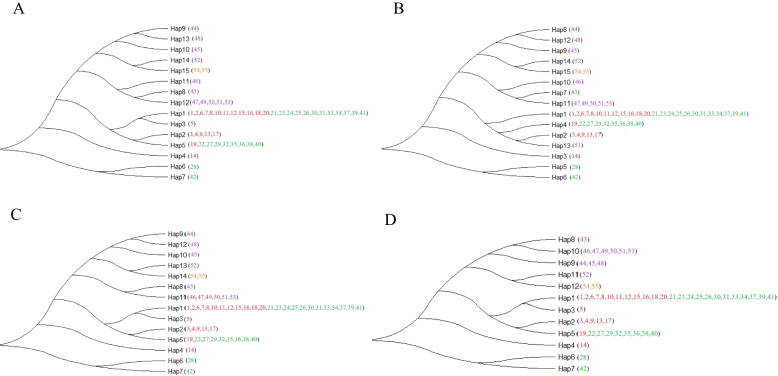
Chloroplast-specific DNA haplotype phylogeny. **A** based on 734 bp chloroplast-specific DNA fragment in length with 205 variable sites; **B** based on 674 bp chloroplast-specific DNA fragment in length with 205 variable sites; **C** based on 574 bp chloroplast-specific DNA fragment in length with 185 variable sites; **D** based on 474 bp chloroplast-specific DNA fragment in length with 130 variable sites. Note: The red numbers represent *A. chinensis* var. *chinensis*, the green numbers represent *A. chinensis* var. *deliciosa*, the purple numbers represent *A.arguta* and the apricot numbers represent *A. polygama*

In Fig. 6, the tree with the highest log likelihood is depicted. Across all four methods of analysis, a consistent finding emerged: one of the most prevalent haplotypes in the examined populations was identified as the major haplotype, denoted as H1. Haplotype H1 was shared by A. chinensis var. deliciosa and A. chinensis var. chinensis, encompassing a total of 24 varieties. In contrast, the remaining haplotypes were characterized as rare and unique. Some haplotypes were exclusive to specific cultivars, occasionally even detected in single individuals. For example, the H15 haplotype was exclusively found in A. Polygama.

**Fig. 7:**
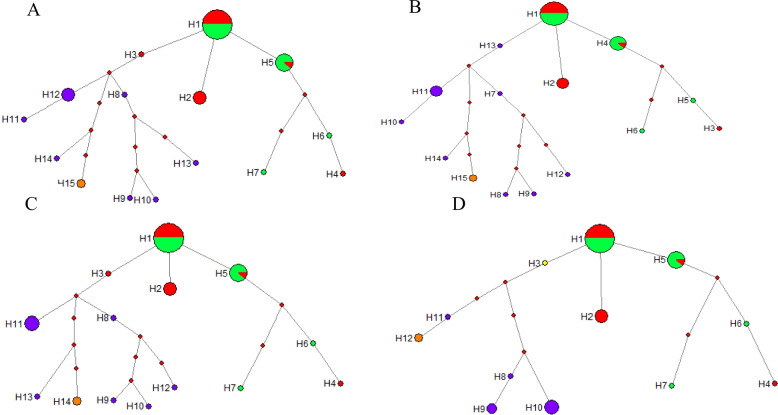
Chloroplast-specific DNA haplotype network. **A** based on 734 bp chloroplast-specific DNA fragment in length with 205 variable sites; **B** based on 674 bp chloroplast-specific DNA fragment in length with 205 variable sites; **C** based on 574 bp chloroplast-specific DNA fragment in length with 185 variable sites; **D** based on 474 bp chloroplast-specific DNA fragment in length with 130 variable sites. Note: The size of the circle with different colors are proportional to frequencies. The red circle represent *A. chinensis* var. *chinensis*. The green circles represent *A. chinensis* var. *deliciosa*, the purple circles represent *A.arguta*, the apricot circles represent *A. polygama*. The red boxes represent the intermediate haplotypes

Figure 7 displays the analysis of haplotype frequencies and their distribution pattern. Haplotype network analysis revealed that three of the haplotypes groups identified based on H1, in which H2 works individually, this suggests that samples sharing haplotype H2, including individuals 3, 4, 9, 13, and 17, all of which belong to A. chinensis var. chinensis, may not have undergone hybridization with other kiwifruit materials in recent generations. In contrast, haplotypes such as H3, H4, H5, and H13 exhibit ongoing evolution.

## Discussion

Differences in the characteristics of nuclear and chloroplast genomes lead to variations in the application focus of different DNA markers. It is vital to conduct an in-depth exploration to determine the most suitable marker for the specific field of research. It can be concluded that SNPs obtained through sequencing offer greater efficiency in population structure studies compared to PCR-based nSCoT markers. This is due to the subjective nature of nSCoT genotyping results. With the increasing availability of next-generation sequencing at affordable costs, the application of SNP-based technology in biology is expected to become more widespread. Among the three types of molecular marker dataset, chloroplast-specific DNA haplotype markers appear to be more effective for measuring population genetic structures. Haplotypes based on genome-wide data may be particularly valuable as functional markers for genetic diversity studies. Chloroplast-specific DNA haplotype phylogeny reveals that A.arguta and A. polygama are closely related to A. chinensis var. chinensis, all of which belong to the category of hairless-peel fruits. This suggests the significance of the trait related to fruit hairiness in kiwifruit classification.

No significant differences were observed between certain cultivated accessions and wild germplasms, indicating that most accessions are derived from the domestication of wild kiwifruit resources. Due to the relatively recent initiation of kiwifruit breeding in China, most of the cultivated kiwifruit varieties are concentrated in A. chinensis var. chinensis and A. chinensis var. deliciosa. Consequently, gene flow appears to be more evident within these varieties. Furthermore, in China, the emphasis during the breeding process has been on selecting varieties with economically desirable traits. As a result, some kiwifruit resources remain underutilized. To continuously integrate different genes into the gene pool of kiwifruit resources, it is necessary to introduce new breeding methods and promote hybridization between species within the genus Actinidia through long-term kiwifruit breeding and production activities. This will help broaden the genetic diversity of kiwifruit plants, enhance their resistance to diseases and flooding, and increase their medicinal value, among other benefits.

## Data Availability

The datasets used or analysed during the current study are available from the corresponding author on reasonable request.

## Abbreviations

cpDNA: Chloroplast DNA
SNP: Single nucleotide polymorphism
nSCoT: Nuclear start codon targeted polymorphism
PPB: Percentage of polymorphic bands
PIC: Polymorphism information content
UPGMA: Unweighted pair group method with arithmetic mean
PCA: Principal component analyses
